# Believing and social interactions: effects on bodily expressions and personal narratives

**DOI:** 10.3389/fnbeh.2022.894219

**Published:** 2022-10-06

**Authors:** Rüdiger J. Seitz, Hans-Ferdinand Angel, Raymond F. Paloutzian, Ann Taves

**Affiliations:** ^1^Department of Neurology, Centre of Neurology and Neuropsychiatry, LVR-Klinikum Düsseldorf, Medical Faculty, Heinrich-Heine-University Düsseldorf, Düsseldorf, Germany; ^2^Institute of Catechetic and Pedagogic of Religion, Karl Franzens University Graz, Graz, Austria; ^3^Department of Psychology, Westmont College, Santa Barbara, CA, United States; ^4^Department of Religious Studies, University of California, Santa Barbara, CA, United States

**Keywords:** belief, trust, narratives, rituals, preferences, valuation, credition

## Abstract

The processes of believing integrate external perceptual information from the environment with internal emotional states and prior experience to generate probabilistic neural representations of events, i.e., beliefs. As these neural representations manifest mostly below the level of a person’s conscious awareness, they may inadvertently affect the spontaneous person’s bodily expressions and prospective behavior. By yet to be understood mechanisms people can become aware of these representations and reflect upon them. Typically, people can communicate the content of their beliefs as personal statements and can summarize the narratives of others to themselves or to other people. Here, we describe that social interactions may benefit from the consistency between a person’s bodily expressions and verbal statements because the person appears authentic and ultimately trustworthy. The transmission of narratives can thus lay the groundwork for social cooperation within and between groups and, ultimately, between communities and nations. Conversely, a discrepancy between bodily expressions and narratives may cause distrust in the addressee(s) and eventually may destroy social bonds.

## Introduction

Believing conveys personal meanings that are constructed by means of perceptual and evaluative processes (Coltheart et al., 2011; Seitz et al., 2017). Believing processes also include predictive coding, which influences peoples’ behavior as they make decisions. Whereas in philosophy beliefs are thought to be consciously held propositions (Stanford Encyclopedia), the concept of *credition* posits that beliefs are based on believing processes that are mostly nonconscious but that may become conscious when a person is believing (Angel et al., 2017). Accordingly, at the neuropsychological level believing can be considered as a higher-order, integrative brain function similar to cognition and emotion (Angel et al., 2017; Angel, 2021). Notably, behavioral studies have revealed that the formation and updating of beliefs occur at a pre-linguistic level in non-human primates (Maravita and Iriki, 2004). However, humans can become aware of their beliefs and express their content and strength verbally (Oakley and Halligan, 2017; Seitz and Angel, 2020). Consequently, beliefs can be expected to play an important role in both verbal and non-verbal social interactions.

The findings and argument of this article lead to a novel perspective of the role of believing and beliefs in the shared realities of cultural dynamics that is underrepresented in recent literature (Kashima et al., 2018). They also raise the intriguing question of how communicating personal statements touches upon the as-yet not well-understood role of conscious awareness of belief contents in transmitting them from one person to other people. In order to explore these and related issues, let us begin with a look at the relationship between information processing and the formation and articulation of beliefs, trusting them, the human capacity to be conscious, and other aspects of human engagement that are rooted in credition–processes of believing. We will then be prepared to conceptualize the role of perceptual information processing, emotional valuation, and their appraisal in believing and decision making. Our discussion then shifts to examining the impact of believing on the generation of bodily expressions and verbal statements—which may be either intended or involuntary but are nevertheless interrelated in social communication. We conclude by describing these aspects of believing processes in relation to the dynamic evolution of social collaborations in ethnic groups, which may also apply to cultures and worldviews.

## Belief Formation, Trust, and Awareness

### Information processing

Living beings process a great deal of information about physical objects in their environment. Importantly, at the neurophysiological level, they process this information very rapidly. This speed of transmission is part of what enabled them to evolve. In the same manner, they also rapidly process information about events, which are things perceived by an observer as a change in the environment with a beginning and an end (Zacks and Tversky, 2001; Asprem and Taves, 2021). The information about objects and events has to be weighed as beneficial or aversive, and must allow a person to react both fast and appropriately. When positive emotions are involved, affirmative beliefs become manifest; this is in contrast to when negative emotions are involved, which render objects and events as threatening, irritating, or disgusting (Seitz et al., 2018). Such processes, which involve the complex interaction of the perception of objects and assessing their positive or negative value and emotional tone, are intimate to meaning-making and remaking (Paloutzian and Mukai, 2017). They constitute the fundamental ground of the processes of believing at the neuropsychological level, and they extrapolate to the psychological, social, and cultural levels as well, with increasing complexity at each step. Therefore, across levels of analysis, a belief is a meaning that has been made and stored in memory (Seitz et al., 2022).

In addition to the pre-linguistic type of belief formation and updating as noted above, humans also process verbal information. From birth onwards, verbal information is provided by caregivers, and later with increasing age by many other people. Also, verbal information is often presented in a ritual fashion through nursery rhymes, songs, fairy tales, and stories. Such narratives are spoken or written accounts of events that are connected and loaded with positive emotions. Often, such narratives can function as the basis for the intuitive generation of conceptual beliefs about a personal self, a family, a social group, and a community, as well as place, time, morals, justice, and many other aspects of social life (Belzen, 2010a, b; Zaidel, 2019). From an evolutionary perspective, it is interesting that ritual activities and play behavior have a number of features in common and are widespread in non-human animals (Mori, 2020).

### Repetition and trusting

People typically believe that what they have perceived is accurate and true; they intuitively trust their perceptions, because they are processed easily and concerning the environment typically are true (Brashier and Marsh, 2020). However, if the events are below 200 ms, and, therefore, cannot be stored in memory correctly, claims about the perception are typically not accurate (Bear et al., 2017). Thus, there is a close relationship between believing and trust. Trust has been defined in different fields of study—personality theory, sociology, economics, social psychology—and summarized as an individual’s belief and willingness to act (Lewicki and Tomlinson, 2014). As such, trust comprises a number of social-cognitive dimensions such as competence, integrity, predictability, compassion, compatibility, etc. (Kappmeier, 2016). For example, it has recently been shown that repetitive stimulation induces people to trust their perceptions, which can lead to an illusory truth-effect (Fazio and Sherry, 2020). These findings suggest that, although someone may believe one or another element of environmental information, it may require a number of converging observations before a person trusts a situation or another person.

### Awareness

The processes of believing occur so rapidly that information perceived from the environment is integrated with internal emotional loadings prior to conscious awareness (Wegner, 2003; Seitz et al., 2009; Park and Tallon-Baudry, 2014). The speed of this integration is similar to that of the generation of a simple motor action; for example, as when the flexing of an index finger is initiated below conscious awareness (Libet, 1985; Hallett, 2016). In a similar way, when developing expectations and preferences humans typically rely on relatively stable conceptual beliefs without being aware of them (Williams, 2020). This finding supports the notion that understanding how unconscious knowledge works is fundamental to understanding human thought processes and mentation more generally (Augusto, 2010).

Even so, the content of thoughts and beliefs may enter conscious awareness and allow an individual to give a verbal account of what he or she believes (Oakley and Halligan, 2017). The neural processes underlying belief formation and updating have been shown to demand a phylogenetic expansion of brain functions that enable people to make verbal statements that begin with “I believe …” (Seitz and Angel, 2020). The ability to express what one believes has been hypothesized to be the prerequisite for auto-reflexive as well as interpersonal belief evaluation (Langdon and Coltheart, 2000; Seitz, 2022). However, most behavior is not pre-thought or generated by “reason”. But as soon as someone becomes aware of an intended action, the person is capable of voluntarily modulating the behavior up to a certain point, as has been shown experimentally (Filipovć et al., 2000). This capability is reflected in the common German expression “sich beherrschen” (keep calm). It means that a person who might spontaneously act with high internal drive in a possibly exaggerated manner has a limited time window in which to calm down and voluntarily suppress aversive acts, so that the behavior turns out to be appropriate for the circumstance. The need for humans and other animals to modulate their actions so that they are consistent with the norms and values of the individual’s social network requires that a valuative process be part of the processes of believing.

## Valuation of Information

### Probabilistic

**Figure 1:**
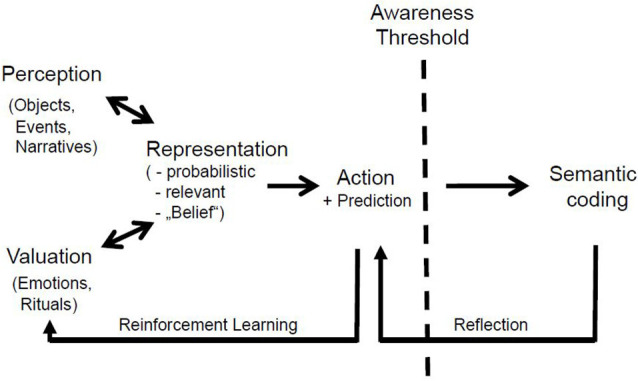
The neural processes underlying believing below and above a person’s conscious awareness. The processes on the left evolve fast, within the range of milliseconds in cortico-subcortical brain structures, allowing for the formation and updating of beliefs and corresponding action generation. Perception pertains to environmental information, whereas valuation mediates the emotional valence. These processes can be the object of empirical neuroscience research. The neural processes on the right occur in the realm of conscious awareness and capitalize on verbalized information which can become the object of a person’s reflection or appraisal. Note that the participation in rituals provides an immediate emotional loading to such stereotypic events that are instructed by corresponding narratives. These putative processes have stimulated epistemic theories in the humanities since antiquity.

When humans interact with objects or other people, they intuitively develop an affective attitude that reflects the putative beneficial or aversive impact of the encounter (Seitz et al., 2009; Prochnow et al., 2013). The resulting probabilistic perceptive-emotional accounts are the basis for the person’s predictions of future events as well asfor context-related adaption of his or her behavior (Figure 1). Accordingly, the emotional valence renders the perceived object or event personally relevant, and shapes what a person intuitively uses for behavioral control. The neural representation that provides this tight neural link between the information that has been perceived and the prediction that determines the selection of a subsequent action has a probabilistic character and, thus, may be considered a consequence of believing processes, i.e., a belief (Seitz et al., 2018). Similarly, narratives about how an individual comes to belong to or be part of a certain group—such as a family, ethnic tribe, or regionally defined group—exert a strong influence when they are presented in ritual acts. And because of their strong affective components, rituals stabilize social behavior within and across generations—a phenomenon that has also been described in non-human animals.

### Speed and affect

In addition to its probabilistic nature, emotionally-laden information may be differentially detected as, e.g., when its speed is in the range of milliseconds (below human conscious awareness). For example, people could not detect fearful-looking faces when they were flashed for 33 ms, but they did detect the faces when presented for 67 ms (Pessoa et al., 2006). Also, in a backward masking task, the occipitotemporal N170 electrical potential was enhanced when people observed facial expressions that were categorized as emotional, which suggests that their brains were processing information from the faces without conscious awareness (Smith, 2012). In another study of how long it takes for someone to detect a fearful face when presented subliminally and supraliminally, it was found that detecting a fearful face occurred at approximately 260–300 ms after presentation (Pegna et al., 2008). This finding corresponds to the notion that perceptual awareness emerges at about 200 ms, with modality-specific negative changes in the brain at 120–200 ms and a later modality-independent positive potential at about 300 ms (Koivisto and Revonsuo, 2010; Marti and Dehaene, 2017; Dembski et al., 2021). The amygdala seems to be of critical importance for this to occur since it is said to coordinate the activity of cortical networks during the early evaluation of the biological significance of affective visual stimuli (Pessoa and Adolphs, 2010). The activity of the amygdala has also been found to be modulated in people who had to look at photos with stimuli placed to the left or right of pictures that depicted emotionally loaded fearful human faces (Straube et al., 2010; Kim et al., 2016). These data suggest that under certain conditions, subconscious processing of inferences may overcome the threshold of awareness, e.g., when there are temporally extended or repetitive observations. In fact, there is evidence that low-level inferences may occur fast and below conscious awareness, whereas high level conscious inferences integrate information across different sensory modalities and larger spatial scales and, therefore, take more time (Olcese et al., 2018).

Emotions shape what a person remembers because emotional cues play a fundamental role in gating relevant information and suppressing non-relevant information. For example, positively valenced stimuli improve prospective memory performance such that events with a strong emotional loading can be retrieved from memory more vividly than neutral events (Hostler et al., 2018; Kensinger and Ford, 2020). In addition, in empirical studies with more than 20,000 individuals, it was found that attitudes based on emotions were relatively fixed and decayed less over time if the emotions were positive (Rocklage and Luttrell, 2021). Thus, the emotional loading of the inherently ambiguous information about objects and events enhances the probability of its encoding as a personal imagination in memory.

### Fluidity

Nevertheless, owing to their probabilistic nature, beliefs are fluid and can be updated upon new evidence depending on the subjective weighing of the previous or new information (Angel and Seitz, 2017; Seitz et al., 2018; Kube and Rozenkrantz, 2021). Even positive perceptions can turn into negative perceptions. An example is the change of attitude towards wetness of the skin. Thermoregulatory behavior is known to depend both on peripheral sensors that communicate their information to the brain, as well as on temperature sensing within the brain (Tan and Knight, 2018). Specifically, individuals were found to perceive warm-wet and neutral-wet stimuli as significantly less wet than cold-wet stimuli on their skin, although the stimuli had the same moisture content (Filingeri et al., 2014). Likewise, on a hot summer day, very wet skin due to a lot of perspiration can cause someone to feel uncomfortable and possibly some disgust, whereas similarly wet skin as the result of a cool bath may be perceived as joyful and refreshing. Yet, on the evening of such a summer day, a bath of similar temperature may be experienced as unpleasant and to be avoided, very much similar to a bath on a cold and overcast day. These examples are consistent with the observation that attitudes, preferences, and values are not absolute. Rather, their coding of valence seems to follow a relative scale (Vlaev et al., 2011; Pischedda et al., 2020).

## Evolving of Valuation

### Early age

Before children begin to speak and learn words for the objects and events around them, they learn to interact nonverbally with other people. They learn to recognize emotional facial expressions and communicative gestures. Thus, children learn to make sense of communicative acts and nonverbal gestures from first-hand observation (Harris et al., 2018). In addition, they imitate the motor acts they observe and learn that they get praise for doing this well (Piaget, 1978). In doing so, they learn to associate their own facial expressions with their emotional feelings. Evidence for this is illustrated by a field experiment in which it was found that young people who reported more intense experiences of fear and happiness were more accurate in recognizing facial expressions of fear and happiness by the early age of 5 years (Buchanan et al., 2010). Children have also been reported to understand the content of other minds through social and communicative interactions with others, which requires that they compare their own perspective to that of others (Tomasello, 2018). As children learn to acquire such information, which comes from multimodal external sources, they apparently reason about how trustworthy the information they are receiving is (Harris et al., 2018). Children thereby develop a sense of trust in their representations of their environment, of which two important aspects are a sense of authorship and causal inference.

### Inferences and conceptual beliefs

It has been shown that humans track the likelihood that their inferences are correct such that probabilistic learning and estimating confidence in what has been learned are intimately related (Meyniel et al., 2015). Although confidence increases with the number of observations, children have been found to be prone to set aside their own prior convictions and defer to informants for social reasons when they are presented with unexpected or counterintuitive but still credible testimony (Harris et al., 2018). Thus, beliefs can be modified in view of new information that is valued higher than previous information. Specifically, social reasoning appears to be valenced higher than one’s own sense of trustworthiness (Harris et al., 2018). It may seem remarkable that personal appreciation of a social relationship is intuitively valued so strongly as to override one’s individual stance. But people in close relationships are likely to be connected by similar beliefs and values, which allows them to maintain common meaning systems (Andersen and Przybylinski, 2018). Such commonality seems to involve predictions about the other person’s most likely behavior, including the non-verbal mentalizing capacity called “theory of mind” (Bird and Viding, 2014). For example, in the cortical areas that have been associated with the “theory of mind”, personally familiar faces have been shown to evoke stronger responses than faces of famous people who happen to be known but not personally (Gobbini et al., 2004). Further, people show an inherent tendency toward intuitive prosociality, as social learning involves areas ascribed to the so-called social brain such as the ventral medial prefrontal cortex, in addition to areas involved in self-relevant learning (Lengersdorff et al., 2020).

**Figure 2:**
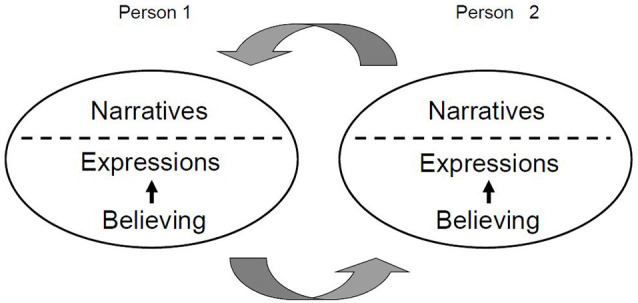
Interpersonal exchange of personally relevant information. Believing can result in bodily, non-verbal expressions and/or verbal statements executed spontaneously below the realm of conscious awareness (dotted line). Narratives include spontaneous personal statements and refined conceptual beliefs. Note that interpersonal interactions involving non-verbal and/or verbal expressions and narratives also occur within groups of people and among members of communities.

Children probably acquire conceptual beliefs in an intuitive fashion from early on. For example, nursery tales, narratives about ritual acts, and the proper prayers in religious families are communicated to children regularly and shape their beliefs and worldviews. Consistent with this, it has been argued that a sense of morality could emerge in a developmental system in which children’s early capacities are shaped by interpersonal engagement (Carpendale and Hammond, 2016). Only later, upon explicit reasoning about such conceptual beliefs, will the information in these communications be brought into conscious awareness so that the person can begin to talk about their beliefs and what their implications mean to them (Figure 2).

## Communicating The Contents of Beliefs

### Mentalizing and self-narratives

Beliefs about objects and events are behaviorally highly important and inadvertently affect bodily movements, as can be observed in facial expressions, gestures, and other coordinated or uncoordinated actions (Dael et al., 2012). It typically seems almost impossible to suppress these spontaneous bodily reactions because their purpose is to immediately convey behaviorally relevant information to others (Figure 2). An example of such a bodily reaction is facial mimicry. Facial mimicry occurs when someone observes the facial expression of an emotion and has a strong impulse to express the same emotion (Müller et al., 2019). Thus, emotionally loaded attitudes and beliefs can facilitate bodily expressions of feelings of which the individual may be unaware. However, people can also become aware of their beliefs and express their content semantically—a process that has been referred to as internal broadcasting (Oakley and Halligan, 2017). Such intrapersonal communication occurs in several modes including inner dialogue and self-talk (Oles et al., 2020). These inner dialogues are characterized by so-called “I positions”, which represent familiar elements of first-person experience (Langland-Hassan, 2021). Such first-person semantic expressions are “I find”, which is primarily emotional, “I think”, which sounds rational but is nevertheless vague, and “I believe”, which conveys a clear, unambiguous personal stance (Seitz and Angel, 2020). Expressions such as “I like …”, “I want …” and “I believe …” may also convey an affirmative attitude. In contrast, verbal expressions that convey aversive information or a negative attitude are “I fear ‥.”, “I hate …”, “I am angry about…”, and “I am disgusted …”. Both the affirmative and the negative meanings of these and similar expressions are stored in long-term memory and can be retrieved at later times. In both cases, the individual entertains either an inclination towards or an aversion to the perceptual-emotional accounts. These internal propositions enable a person to perform abstract thought and executive functions, and thereby support meta-cognition.

Narrating our personal past connects us to ourselves, our families, our communities, and our cultures (Fivush et al., 2011). Conceptual beliefs as expressed in personal narratives are comprised of information about autobiographical memory that underpins constructs of personal self and agency that were created in non-conscious systems (Oakley and Halligan, 2017). Thus, the so-called narrative or autobiographical self represents a self-image that consists of memories of the past and intentions about the future, constituted from the various stories that people have been told (Gallagher, 2000). Based on this, self-talk supports self-reinforcement, self-management, self-criticism, and social assessment (Oles et al., 2020; Paloutzian et al., 2021a). In other words, humans are in the position to reflect upon the contents of their beliefs (Figure 1). Evaluation of such internal narratives help someone assess the degree to which they reflect reality and are trustworthy, and to modify them with respect to relevant environmental conditions. An implication is that people can reflect on their behavior and act for reasons they can specify.

### Honest or not?

Communicating to others *via* narratives may be intended to convey personally relevant information in order to enhance interpersonal relationships, possibly for the sake of socio-ecology allowing for common goals or actions (Romano et al., 2021). We tend to perceive someone as authentic when the person’s verbal reports and spontaneous bodily expressions including the facial expressions are congruent with each other (Franz et al., 2021). Then, we tend to trust what the person says. However, someone with a manipulative or deceptive intent can covertly modify the narrative as detailed in the truth-default theory (Levine, 2022). In these cases, the speaker’s spontaneous bodily expressions may convey an intent that differs from his or her statements or narratives. If the recipients of the communication detect such a discrepancy, they may perceive the speaker’s message as false and the person as unreliable. In particular, a delay and discrepancy between the speaker’s thought-based verbal expressions and spontaneous bodily expressions may signal that the speaker is anxious, unsure, thoughtless, or deceitful. Concerning narratives, there is an intriguing question about whether emotional valence is communicated by the vocal tuning of verbal expressions, or is conveyed by words themselves. On this issue, a correlation analysis of the assessment of more than 1,400 English words found that abstract words receive higher ratings for affective associations, including valence and arousal, than concrete words (Vigliocco et al., 2014). This finding supports the notion that abstract words are more emotionally valenced than concrete words (Montefinese, 2019). Likewise, language metaphors probably covertly influence people’s reasoning even when different options of how to phrase something are available (Thibodeau and Boroditsky, 2013).

Although narratives evolve rapidly, they can extend over variable lengths of time, depending on the complexity of their content. Neural processing evolves fast enough to allow for the information to be decoded. For example, in audiovisual recognition of vowels coordinated oscillations in speech areas including the inferior frontal gyrus can be detected (Lange et al., 2013). The neural activity changes in relation to the complexity of semantic tasks. For example, it was found recently that neural oscillations encoded endogenously generated linguistic content that surpassed exogenous stimulus-driven timing and rhythm information (Kaufeld et al., 2020). This finding is consistent with the notion that these bioelectric changes could reflect computations related to how humans and other animals infer structure and meaning from acoustic signals (Kaufeld et al., 2020). In other words, the data suggest that the human brain is capable of creating a meaning from a sequence of acoustic stimuli that goes beyond a single stimulus. This may be the basis for abstraction, which allows us to make “bigger” meanings out of the initially specific meanings, i.e., in conceptual psychological terms, to make more global meanings out of lesser situational meanings (Park, 2010). This is also of relevance to the notion of transcendence in language processing (Mesulam, 1990).

## Individuals in Groups

### Identity and bonding

Groups allow their members to behave differently (Barrett et al., 2001). For example, primates of the same species do not necessarily act in an identical way to all members of their species. But they do demonstrate in-group bias, favoritism, and altruism, as well as out-group prejudice, disfavor, and lack of help of an “other”. This pattern of behavior is identical to what Tajfel described in elaborating the social identity theory (Outten et al., 2018). In classic research, when subjects (children, adults, teenagers, adults) originating from different cultures are randomly divided into groups (and know that they are assigned to their group by random chance), they still show an in-group bias and out-group prejudice (Tajfel, 1981). The in-group (“Us”) is better, smarter, prettier, and the out-group (“Them”) is stupid, worse, and more ugly. Such findings suggest that primate sociality has evolved so that it is based on bonded social relationships (Dunbar and Shultz, 2007). Bondedness is an explicitly emotional experience that integrates group perception with an internal affective state or intuitive emotional valence. This is related to what has been called relational beliefs (Seitz and Angel, 2020), i.e., a person who feels emotionally connected to another person or persons spontaneously tends to trust them.

An integrated trust model unifies the existing literature on the multidimensionality of trust, and allows us to explore the role of trust in social collaboration as well as the bases of intergroup conflict or tension, as illustrated in ethnic discrimination (Kappmeier et al., 2019). It also is consistent with Tajfel’s social identity theory and can account for the reactions of majority group members towards minorities in different societies (Outten et al., 2018). There is also a close relationship between social identity and the impact of collective memory such that their combined residue can foster either intra-group trust or inter-group conflict (Kappmeier and Mercy, 2019). Thus, someone in Group A claims to have “owned” or “created” this desired object but believes that the “other” in Group B deserves only that undesired thing. Likewise, social comparison processes that result in attitudes and behaviors of the “I am better than you” sort are manifest not only in competition for staples like food and sex, but also in competition for the sake of social recognition and the superiority of oneself within one’s own group. These processes occur without necessarily being clear or explicit to an individual.

In accordance with these intuitive processes, narratives have been stated to support social and cultural structures (Oakley and Halligan, 2017). Narratives can convey the contents of similar conceptual beliefs such as family, honesty, fraternity, equality, charity, etc. to different people. Because narratives are stored in the memory of individual subjects and can be retrieved from their memory at later time points (Seitz et al., 2022), they are fundamental for the conservation of conceptual beliefs in social groups and societies. Narratives provide the reason for and occasion to engage in ritual acts that are practiced in families, social groups, and communities (Schnell, 2012; Gelfand et al., 2020; Mori, 2020). People thereby develop their social identity narratives of ethnic culture as well as individual self-concept (Knight et al., 2018). Concurrent with this, children are taught and learn how to behave in their social environment.

### Cultures and values

Religions are, amongst other things, cultures (Cohen, 2009). One observation consistent with the above argument is that there is an association between the profession of religious devotion and greater trusting behavior (Norenzayan and Shariff, 2008). This association may occur as beliefs in a morally concerned god may stabilize prosocial norms within the culture even in the absence of social monitoring mechanisms. Such stabilizing may occur at the neural level, in that religious beliefs were found to activate regions within a network related to mentalizing of intent and emotion, abstract semantics, and imagery (Kapogiannis et al., 2009). In related research, the comparison of religious and non-religious subjects did not reveal any differences in these activations—in accordance with the notion that religiosity is integrated into cognitive processes and brain networks used in social cognition (Boyer, 2003). Extending the above notions, there are potent models to explain how ethnic views expand among groups and extrapolate to explain the acquisition of similar views and subsequent related behavior in other cultures (Galesic and Stein, 2019). Even so, it is possible for someone’s cultural orientation to change over time as a function of their experiences with and membership in multiple groups, in addition to their normal age-related developmental changes (Knight et al., 2018).

In any case, language is considered to be an inadequate medium for describing inner emotional experiences and communicating them (Dunbar and Shultz, 2007). The reasons are twofold, namely that the speaker needs to become aware of his/her emotional experience and needs to know how to express this experience clearly in words. These descriptions of personal experience also need to have some meaning for the listener. Such communication of meaning is probably straight-forward for basic emotions like happiness, sadness, anger, and fear. However, more complex feelings or “higher” emotional values like empathy, forgiveness, and altruism may not be easily understood or straight-forwardly shared spontaneously. Instead, they may need to be explained by more elaborate verbal descriptions or perhaps be accompanied by a positive emotional descriptor. In this context the concept of shared reality is important. There is lot of evidence that communicators fine-tune their statements in an effort to align them with the attitudes of those to whom they are speaking. Doing this in turn shapes their recall (Higgins et al., 2021) and has been said to promote interpersonal closeness and epistemic certainty.

Extending the above argument further, certain moral values may be considered as higher order emotions and may function in a way similar to them. For example, the feeling of empathy is highly value-laden and implies accepting another person and his or her difficulties in a manner similar to accepting oneself. A common illustration is the moral values codified in religions, such as the Ten Commandments, which provide a guideline for how to behave properly. These and similar teachings are transmitted among people across generations and reflect not only stable language use but are also suited to guide certain behavior according to their norms. Thus, people can reflect on their thoughts, wishes, and actual actions in light of these norms, and thereby become responsible for their actions. At the neural level, a study in which the participants viewed scenes evocative of moral emotions showed that the orbital and rostral medial prefrontal cortex and the cortex along the superior temporal sulcus are involved in mediating the above noted value-related events (Moll et al., 2002). Processes such as trusting, forgiving, and believing matter because humans make attributions about these properties and respond accordingly (Paloutzian et al., 2021b). Problems arise between parties when there is an inconsistency between what one says and what one does when verbal behavior and overt actions are discrepant (ibid). Collaboration can only re-start in small, reciprocal, trust-inducing steps (ibid). This means that the actual experience with the counterpart matches what he or she believes about the counterpart being of particular relevance for international and cross-cultural issues (Schoorman et al., 2007).

## Discussion

The neural processes that afford belief formation, believing, and the updating of beliefs occur spontaneously in the time domain of milliseconds. As summarized in Figure 1, belief formation includes the integration of information coming from the environment and attribution of emotional value, with both aspects resulting in personal probabilistic representations. This model accounts also for the formation of socially adaptive beliefs that are sensitive to social rewards and punishments (Williams, 2020). Accordingly, beliefs are intimately coupled with subjective experience prior to complex processing of prediction of a behavioral outcome and to awareness of the incoming information as suggested recently (Key et al., 2022). Belief updating occurs by means of reinforcement learning *via* cortico-subcortical circuits when actual and predicted information match, whereas new information of high subjective relevance is able to induce a change in the belief (Figure 1). Therefore, the cerebral networks that are involved allow for the storage of beliefs in memory (Seitz et al., 2022). This is consistent with the notion that cerebral representations are memories that are localized in neural networks and, when activated, enable access to this stored information (Wood and Grafman, 2003).

In a very similar hypothesis, experienced events were labeled as event knowledge (Taves and Asprem, 2016). Even though event recognition and other processes are occurring, most brain processes are not accompanied by any discernable changes in subjective awareness (Halligan and Oakley, 2021). But people can retrieve stored information from memory, whereby it then enters their conscious awareness (Figure 1). This retrieval is a critical prerequisite for a person to be able to semantically phrase what he or she is believing. This can typically be done by implicit or deliberate self-talk or a prayer. Either way, people can reflect on their beliefs and sharpen their awareness of the information. This reflection probably corresponds to the notion of the belief evaluation systems (Coltheart et al., 2011; Sugiura et al., 2015), which may be explained by invoking the concept of event models. Event models are constructed from the point of view of the person who perceives the entities and functional relations involved in understanding a specific state of affairs (Radvansky and Zacks, 2017). As a result, they contain information that the person considers relevant regarding spatiotemporally located entities (agents and objects) and establish the structural and linking relations between them as he or she understands them in light of their previous experience [i.e., in light of plausible types of events (event schemas) and their own particular memories of past events (other event models)]. Also, relations that link objects and events, which include the causes and consequences of events, play a crucial role in the way the model is structured, linked to other events, and retrieved on later occasions. In fact, upon reflection people can modify their behavior so that it deviates from the predictions based on beliefs only. Beyond that, belief evaluation enables humans to communicate what they believe to other people (Oakley and Halligan, 2017). Consequently, exploring the neural principles of belief formation and updating is central to the research discipline of social cognitive neuroscience (Lieberman, 2010).

In the concept of credition, believing is a fundamental brain function that links emotional valence to sensory perceptions, rendering them personally relevant and memorable (Angel et al., 2017; Seitz et al., 2018, 2022; Seitz and Angel, 2020; Angel, 2021). In fact, emotion signals have been shown to enhance processing efficiency and competitive strength of emotionally significant events through gain control mechanisms mediated in the amygdala and interconnected prefrontal cortical areas (Pourtois et al., 2013). By this means, emotions become fundamental to the self-regulation of behavior (Peil, 2014), although they may change over one’s lifetime. For example, toys, food, and drinks that infants and children love can be undesirable to adults. Conversely, the personal relevance of objects or events can be modified by diseases. For example, patients handicapped by a disabling disease of the body may still have a positive perspective on life in a way that may seem impossible for a healthy person. Thus, valence may inadvertently be changed by external events, which can result in an update or even dismissal of a hitherto held belief (Angel and Seitz, 2017). Moreover, brain diseases leading to neuropsychological deficits and psychopathological disorders have been shown to result in the formation of abnormal beliefs that can cause inadequate or even aversive behavior which can undermine social bonds (Connors and Coltheart, 2011; Seitz, 2022).

Probably because of the emotional and rapidly evolving nature of underlying neural processes, the processes of believing take place below a person’s awareness and, thus, outside his/her reach. This becomes obvious in social interactions in which a person judges his/her counterpart and *vice versa*. Humans are known to rapidly develop an intuition or belief about whether to trust another person and how to react to him/her (Potthoff and Seitz, 2015). Such primal beliefs influence our spontaneous bodily expressions, as has been found in facial mimicry and bodily movements (Figure 2). Both are expressions of non-verbal communication (Dael et al., 2012). However, beliefs may enter conscious awareness—probably in a graded fashion rather than in an all or none manner for the different sensory modalities. Their content then can be phrased verbally, rehearsed internally, and communicated as narratives to others (Figure 2). It is a specific human capability that narratives underlying conceptual thinking and believing cannot only be transmitted *via* speech but can also be written down and transferred to other people as scripts, letters, or books (Belzen, 2010a, b). Such documents can be read, reflected on, and re-read, allowing for new associations and novel creative thoughts. Written concepts also support social memory. Thereby, narratives turn out to be fundamental for the autobiographical self and the formation of social groups.

As to the bases for making predictions from such narratives, humans are in a position to explore whether their actions concur with norms, rules, and expectations of other people or whether they offend them. Having these options corresponds to what has been called to act based upon reasons (Proust, 2003). In so doing, people become “responsible” for their actions. This does not exclude that they may flexibly manipulate group-mates’ behavior to tactically deceive them, as has been shown in experimental food competitions in primates (Hall and Brosnan, 2017). Humans may also intentionally deceive people, such as when there is a discrepancy between their pre-thought verbal statements and their spontaneous motor expressions. In this connection, there are neurophysiological and neuroanatomical bases for cognitive and affective theory of mind, with interpersonal and intrapersonal dimensions that humans can use to determine when cheaters need to be punished (Westby, 2014). In fact, humans are highly capable of detecting whether someone’s verbal and non-verbal communication are consistent or inconsistent with each other. For human behavior, these different possibilities are accounted for by the cultural brain hypothesis. This hypothesis posits that brains have been selected by evolution for their ability to store and manage information that was acquired through social and asocial learning (Muthukrishna et al., 2018). Consistent with this idea, many components of language, including extra-linguistic meaning systems and the communication of symbolic meaning, have neurobiological roots that go back millions of years in evolutionary time (Zaidel, 2019).

The data are consistent with the notion that our capacity to use language creatively enables us to gain awareness of the mental worlds of other people, and that we can communicate our own imaginative play, creative narratives, original thoughts, arguments, and feelings to them (Markl, 2002). In essence, the multi-level analysis presented in this article appears capable of bridging the gaps between the level of neural systems to the behavioral level in individuals to the social level. We assume that probabilistic processes at the neural level and increased probability in a stepwise fashion as we go up to the behavioral and social levels. Nevertheless, it is important to remember that the relations between symbols and content can be quite variable across different cultures. For example, the association of *white* with *joy* and *black* with *grief* is a Western tradition, with associations in the opposite directions in Asia. Also in Western communities, shaking one’s head means “no” and nodding means “yes”, but these head movements convey opposite meanings in other cultures. Similarly, there are complex patterns of language evolution with respect to different ethnicities that involve adopting, keeping, and replacing vocabularies and grammars (Das et al., 2016). Relatedly, ratings of the degree of affect in neutral faces have been shown to not be neutral; they are instead loaded with different levels of ambiguity, and thus may yield important differential psychological consequences (Schneider et al., 2016). These findings raise interesting issues (yet to be solved) about the concurrence, discrepancy, and ambiguity of our verbal and non-verbal communication. In any case, human intelligence appears to be a combination and enhancement of properties found in non-human primates including mentalizing (theory of mind), imitation, and learning from verbal testimony (Roth and Dicke, 2005; Harris et al., 2018).

There is much psychological evidence that supports the proposition that majority views are held with stronger confidence and expressed more quickly than are minority views, regardless of any social pressure to conform (Koriat et al., 2016). Thus, social consensus plays a causal role in supporting and enhancing a person’s confidence in beliefs, opinions, and attitudes (ibid). Further, social influence is involved when one attempts to either gain social approval or avoid social isolation. But when we consider real-world groups and the issues between them (men vs. women, blacks vs. whites, Middle-Eastern Muslims vs. Western Jews, and Christians, …. the list is endless), with actual fighting and lethal confrontations, we can understand not only why there is intergroup conflict but also how the tendency humans have towards outgroups has its roots in our genetic makeup from eons of evolution (Paloutzian et al., 2021b). Even so, if we humans can evolve inclinations to trust, including trusting our enemies (in graded mutual and reciprocal steps, so that it is possible for the process to work), we may evolve out of group conflict as “built in” to our genes towards contact and collaboration with all humans as one group, so that everybody can love everybody instead of being afraid of them. Ultimately, if everybody would just sit down and talk about their processes of believing, we would learn that we are more ike each other instead of the various ways that we differ.

Quite unexpectedly, it was found that affective content is highly relevant in abstract thoughts and conceptual beliefs (Montefinese, 2019). For example, religious beliefs have been shown to be maintained by prayer and ritual acts but not by deductive or inductive reasoning (Atran and Norenzayan, 2004; Feierman, 2009). Today, many people have greater confidence in their scientific beliefs than in their religious beliefs, although similar patterns of justification have been described for both kinds of believing (Harris and Corriveau, 2020). However, the comfort and support provided by religious organizations may grow when people experience more harshness, when coping resources begin to diminish, and when environmental pressures demand a greater effort (Seryczynska et al., 2021). Because the adults’ perception of the relation between religion and science is heavily shaped by their sociocultural contexts, the relation between religiosity and the valuation of science varies profoundly between different countries (Payir et al., 2021). This does not preclude that the contents of different beliefs, such as political or religious beliefs, may be reported to be equivalent but not identical (Oviedo and Szocik, 2020).

In conclusion, the notions of belief and believing are complex cognitive constructs similar to culture and consciousness that may be amenable to naturalistic exploration in an evolutionary framework (Singer, 2019).

## Data Availability Statement

The original contributions presented in the study are included in the article, further inquiries can be directed to the corresponding author.

## Author Contributions

RS: designing, drafting, and editing. H-FA, RP, and AT: drafting and editing. All authors contributed to the article and approved the submitted version.

## Funding

This work was supported by Frontiers Science Publisher.
